# The Functional Vision Score—A New Method to Assess Visual Function

**DOI:** 10.1155/joph/9989956

**Published:** 2026-04-01

**Authors:** Veronika Yehezkeli, Eden Hollander, Shani Shaulian, Elad Moisseiev

**Affiliations:** ^1^ Department of Ophthalmology, Meir Medical Center, Kfar Saba, Israel, mmc.org.il; ^2^ School of Medicine, Tel Aviv University, Tel Aviv, Israel, tau.ac.il

**Keywords:** assessment, functional vision, low vision, score, visual function

## Abstract

**Purpose:**

To design and investigate a functional vision test to evaluate visual functioning in a wide range of daily activities.

**Methods:**

Sixty participants were included and divided into 4 groups of 15 each, according to the BCVA in their better eye: 20/20 to 20/25 (Group 1), 20/32 to 20/64 (Group 2), 20/100 to 20/200 (Group 3), and lower than 20/400 (Group 4). A 10‐item test simulating activities of daily life was designed for the study, to evaluate patients’ functional vision. Tasks were evaluated for successful completion and timed. For each task, the time to completion was divided by the mean of Group 1 participants, and the resulting ratio was scored. The total scores of all 10 tasks were termed the Functional Vision Score (FVS).

**Results:**

The mean FVS was 12.5 ± 5.3, 21.8 ± 5.8, 35.0 ± 15.0, and 48.3 ± 15.2 in Groups 1–4, respectively (*p* < 0.001), with a significant correlation between participants’ FVS and the BCVA in their better eye (*R* = 0.79, *p* > 0.001). On average, compared to Group 1, mean times of task completion were 1.5–2 times longer in Group 2, 2–4 times longer in Group 3, and 4–6 times longer in Group 4.

**Conclusions:**

The FVS is simple to perform and assesses vision‐related functionality, enabling a more comprehensive evaluation of visual function. It considers motor and cognitive abilities and not only visual acuity. It may serve clinicians and patients, as well as official agencies, who need a tool to determine functional vision.

## 1. Introduction

Visual function has numerous aspects, including acuity, visual fields, color vision, dark adaptation, contrast sensitivity, and stereopsis. In clinical practice, visual function is usually evaluated based on visual acuity, followed by visual fields. Although these are measures of visual function, they do not necessarily reflect patients’ functional vision. Functional vision is important for numerous activities of daily life and is directly related to patients’ overall ability to function, independence, and quality of life. Impaired functional vision has considerable implications for patients’ occupations and social life. Patients with low vision suffer from decreased quality of life [1].

There is no validated test to measure or evaluate functional vision. Therefore, visual acuity and visual fields are commonly used as surrogates for this important parameter, with a wide range of assumptions between them and their associated level of visual function. For example, the United States Social Security Administration defines “statutory blindness” as visual acuity less than 20/100. Other US agencies define “legal blindness” as less than 20/200, and the World Health Organization defines “blindness” as visual acuity below 20/400 [2]. The International Classification of Functioning, Disability and Health provides a standard language and a conceptual basis for the definition and measurement of health and disability. However, it lacks specific, detailed reference to people with visual impairments [3]. Functional vision is seldom evaluated clinically, and when it is, the assessment is typically based on self‐reporting by patients on questionnaires such as the Visual Function Index 14 (VFI‐14) or the National Eye Institute 25‐Item Visual Function Questionnaire (NEI VFQ‐25) [4–6].

The purpose of this observational study was to design and investigate a functional vision test in order to evaluate visual functioning in a wide range of daily activities and to establish its correlation with visual acuity.

## 2. Methods

### 2.1. Participant Selection

The protocol for this observational comparative study was approved by the Institutional Review Board of Meir Medical Center. All participants were recruited from the patient population of the department of ophthalmology at the medical center from January 1, 2022, to June 30, 2022. All participants were over the age of 18 years and provided signed informed consent prior to inclusion.

The following data were recorded for each participant: age, sex, ocular history, lens status, best‐corrected visual acuity (BCVA), and refraction in each eye. Inclusion criteria required stable visual acuity for at least 1 year prior to enrollment in the study. Patients who were undergoing treatment or who had recent surgery that affected their visual acuity were not included. Additional exclusion criteria included cognitive impairment, motor disabilities and hearing loss, since these conditions could affect the performance of the functional tasks included in the study.

### 2.2. Study Groups

Participants were divided into 4 groups based on the BCVA in their better eye. Group 1 included participants with BCVA from 20/20 to 20/25, Group 2, 20/32 to 20/64, Group 3, 20/100 to 20/200, and Group 4 had BCVA less than 20/400. Participants in Group 4 were all recognized as legally blind by the Israeli Ministry of Health.

### 2.3. Study Design

All participants completed a 10‐item test that was developed for the purpose of this study. The test included 10 tasks that simulated daily functional activities whose performance is based on vision. These visual function tasks were identifying paper currency, locating a specific room in a corridor based on its sign, finding a product in a refrigerator, detecting an object in a room, reading a newspaper headline, differentiating between products of similar shape and size, typing a code into a smartphone, identifying a warning sign, identifying a medication, and filling out a medical form. Participants performed the tasks after receiving an explanation from a researcher. A detailed description of the test tasks is provided in Table 1. Participants performed the tasks using refractive correction (e.g., glasses or contact lenses) that enabled their BCVA.

**TABLE 1:** Description of visual function tasks.

Task no.	Detailed description
1	Bills identification: Participants were given 1000 NIS, and they were asked to give back 270 NIS. Four different bills were used each time, including 20 NIS, 50 NIS, 100 NIS, and 200 NIS.
2	Room location: Participants were asked to identify a specific room in a corridor by its sign. All participants started this task at the beginning of a corridor with many doors on both sides and were asked to find a specific door.
3	Refrigerator: Participants were asked to take a specific product from the refrigerator. Participants were instructed which product to take before opening the door of the refrigerator, not knowing where it was.
4	Object location: Participants were asked to detect an object product in an unfamiliar room. A red box was placed in an unfamiliar room, and participants were asked to find it.
5	Newspaper headline: Participants were given a newspaper and asked to find and read aloud a specific article headline.
6	Product recognition: Participants were given 4 cans of similar size and shape but different food products and labels and asked to choose and give the examiner a specific can.
7	Smartphone code: Participants were asked to insert a 4‐digit code into an electronic device (smartphone).
8	Medication recognition: Participants were presented with 4 different labeled pill packages and asked to give the examiner a specific medication that was asked for.
9	Form filling: Participants were given a routine medical questionnaire and were asked to read it and fill in the indicated blanks.
10	Warning sign: Participants were asked to walk down a corridor, in which a large warning sign was placed. At the end of the corridor, they were asked if they noted it.

*Note:* All tasks were timed.

The participants’ performance was monitored by an observer, and for each item, a score of 1 was given if the task was completed and 0 if not. The time to complete each task (in seconds) was recorded.

### 2.4. The Functional Vision Score (FVS)

After completing the test, each participant received an overall score of 0–10 based on the number of tasks that were successfully completed. In order to evaluate the length of time the tasks took to complete, for each task completed by Groups 2–4, the measured time was compared to the mean time the same task took for participants in Group 1. The times Group 1 participants took to complete each task were used as a standard of comparison for Groups 2–4. The ratio between the measured time and the mean time of Group 1 was defined as the score for that task. Tasks that were not completed were scored as 10 and defined as failures (this was set arbitrarily, since the slowest times recorded for completed tasks by participants in Group 4 were up to 9 times longer than the mean times recorded for Group 1). The total score for the 10 tasks was defined as the FVS for each participant. The maximum possible FVS score was 100, based on failures in all 10 tasks.

### 2.5. Statistical Analysis

For statistical analysis, all visual acuity values were converted to the logMAR scale. According to Holladay [7] and the University of Freiburg study group results [8], no light perception was set at 0.00125/2.9 (decimal/logMAR), light perception at 0.0025/2.6, hand movements at 0.005/2.3, and counting fingers at 0.014/1.85.

Participants’ demographic characteristics were compared using descriptive statistics. *T*‐tests were used to analyze associations between continuous parameters. Pearson correlations were used to determine correlations between parametric variables. One‐way analysis of variance was used to compare parameters between the four groups, such as the times to complete the tasks, and the FVSs.

The minimum sample size was calculated using ANOVA, assuming a large difference (*f* = 1) between groups. This value was used as the FVS was designed to emphasize the differences between groups (the actual differences were even larger). Using a significance level (*α*) of 0.05 and a power of 0.8, for 4 groups, the recommended size was 16 patients each. With a total of 60 patients, the power was calculated as 0.99.

The statistical significance level was set at 0.05. Data were analyzed using SPSS for Windows, Version 25 (IBM Corp, Armonk, NY).

## 3. Results

The study included 60 participants—15 in each group. These included 33 women and 27 men, with a mean age of 60.6 ± 17.9 years (range 21–93 years). Participants in Group 1 were significantly younger than those in the other groups (*p* > 0.001). There was no significant difference between the ages of the participants in Groups 2–4 (*p* = 0.48). Rates of pseudophakia were significantly higher in Groups 3 and 4 than in Groups 1 and 2 (*p* = 0.001). Details of participants’ characteristics are provided in Table 2. Participants in Group 1 had no prior ocular history, while those in Groups 2–4 had a variety of causes for their limited visual acuity. A summary of the participants’ ocular pathologies and BCVA is provided in Table 3.

**TABLE 2:** Comparison of the baseline characteristics of participants in Groups 1–4.

	**Group 1**	**Group 2**	**Group 3**	**Group 4**	**p** **value**

Age (years)	42.5 ± 14.72	66.93 ± 12.91	63.8 ± 18.11	70.33 ± 12.18	< 0.001^∗^
Gender, male, *n* (%)	7 (46.66%)	7 (46.66%)	8 (53.33%)	6 (40%)	0.71
Pseudophakia, *n* (%)	2 (13.33%)	2 (13.33%)	9 (60%)	7 (46.6%)	0.001^∗∗^
BCVA of the better eye logMAR (Snellen)	0.02 ± 0.03 (20/20)	0.31 ± 0.08 (20/40)	0.74 ± 0.14 (20/110)	1.43 ± 0.19 (20/538)	< 0.001
Spherical equivalent of refraction in better eye, D	−0.9 ± 1.84	−1.16 ± 3.35	−0.67 ± 2.02	−0.52 ± 2.01	0.57

^∗^Participants in Group 1 were significantly younger than those in Groups 2–4.

^∗∗^Participants in Groups 1 and 2 had lower rated of pseudophakia than those in Groups 3 and 4.

**TABLE 3:** Distribution of main causes for vision limitation of participants in Groups 1–4.

	**Group 1**	**Group 2**	**Group 3**	**Group 4**

Cataract, *n* (%)	0 (0%)	10 (66.6)	3 (20)	1 (6.6)
Glaucoma, *n* (%)	0 (0%)	5 (33.3)	1 (6.6)	1 (6.6)
Retinal diseases, *n* (%)	0 (0%)	3 (20)	8 (53.3)	10 (66.6)
Amblyopia, *n* (%)	0 (0%)	0 (0%)	1 (6.6)	1 (6.6)
Corneal opacity, *n* (%)	0 (0%)	0 (0%)	0 (0%)	1 (6.6)
Neuro‐ophthalmological disorders, *n* (%)	0 (0%)	1 (6.6)	2 (13.3)	1 (6.6)

*Note:* Participants could have more than one ocular pathology causing visual limitation.

### 3.1. Analysis of Visual Function Tasks

All participants in Group 1 completed all 10 functional tasks, with a mean success score rate of 10.0 ± 0.0. Total scores in the 10 tasks were 9.8 ± 0.3 in Group 2 (two participants completed 9 tasks), 9.6 ± 0.6 in Group 3 (four participants completed 9 tasks, and one only 8), and 8.8 ± 1.1 in Group 4 (only 4 participants completed all 10 tasks, 7 completed 9 tasks, 1 completed 8 tasks, and 3 participants completed only 7 tasks). Despite relatively high rates of task completion in all groups, the differences between groups were statistically significant (*p* < 0.001).

Several tasks showed considerable differences between groups. For example, while all participants could identify monetary bills, filling out a form was successfully completed by all participants in Group 1, 93.3% of participants in Group 2, 80% of participants in Group 3, and only 33.3% of participants in Group 4. A warning sign was noticed by 100%, 93.3%, 80%, and 67.7% of participants in Groups 1–4, respectively.

For each task, mean times until completion were significantly different between groups, with the fastest times in Group 1 and the slowest in Group 4 (*p* > 0.001 for all 10 tasks; Table 4). On average, compared to Group 1, mean times to task completion were approximately 1.5–2 times longer in Group 2, 2–4 times longer in Group 3, and 4–6 times longer in Group 4. Examples are provided in Videos 1 and 2.

**TABLE 4:** Mean ± SD of the times each task took to complete in Groups 1–4.

Task	Group 1 (s)	Group 2 (s)	Group 3 (s)	Group 4 (s)
Identify bills	10.1 ± 7.6	14.3 ± 6.1	26.5 ± 19.8	37.0 ± 26.8
Room location	22.7 ± 3.9	31.1 ± 10.2	39.2 ± 10.6	47.2 ± 10.1
Refrigerator	5.1 ± 4.2	7.3 ± 4.2	17.6 ± 13.6	29.4 ± 24.3
Object location	6.9 ± 2.7	13.4 ± 8.7	21.2 ± 10.9	27.8 ± 13.0
Newspaper headline	37.6 ± 33.3	73.9 ± 72.5	119.0 ± 68.1	140.2 ± 127.2
Product recognition	2.7 ± 2.5	6.1 ± 3.4	12.0 ± 11.8	17.0 ± 6.3
Smartphone code	3.6 ± 2.2	6.6 ± 3.5	14.1 ± 10.8	17.4 ± 15.2
Medication recognition	5.5 ± 3.0	12.2 ± 11.2	20.5 ± 10.9	28.9 ± 12.0
Form filling	44.5 ± 25.8	75.3 ± 25.9	113.4 ± 49.9	119.6 ± 65.4
Warning sign	6.6 ± 4.5	15.4 ± 23.5	26.9 ± 38.0	34.0 ± 41.2

### 3.2. Analysis of FVSs

**FIGURE 1:**
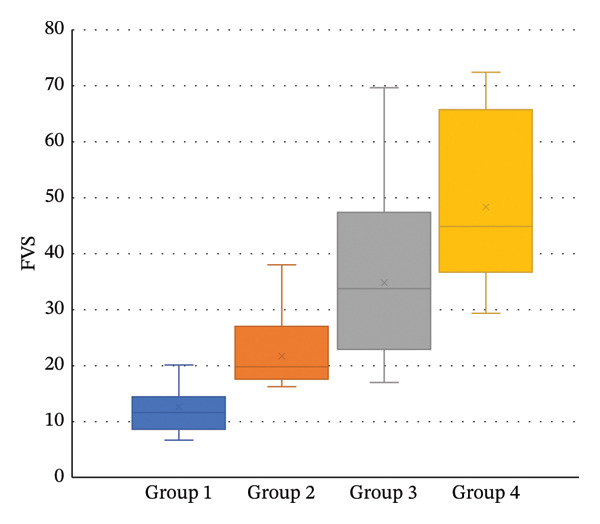
Box‐and‐whiskers plot showing the Functional Vision Scores of Groups 1–4. The top and bottom lines of the box indicate the first and third quartiles, the central line represents the median, and X marks the mean. The ends of the lines are the maxima and minima.

**FIGURE 2:**
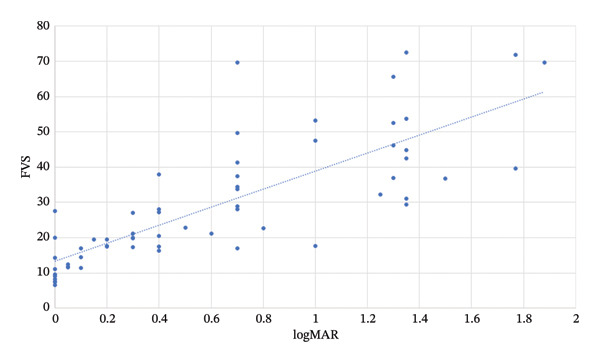
The distribution of BCVA and Functional Vision Scores of all participants demonstrated a strong positive correlation. The dotted line illustrates this correlation, which by linear regression corresponds to the formula: FVS = 25.5 × logMAR + 13.3.

The mean FVS was 12.5 ± 5.3 s, 21.8 ± 5.8, 35.0 ± 15.0, and 48.3 ± 15.2 s in Groups 1–4, respectively (*p* < 0.001). The distribution of the FVS for all groups is presented in Figure 1. A significant correlation was found between participants’ FVS and the BCVA in their better eye (*R* = 0.79, *p* > 0.001). This correlation is presented in Figure 2. Linear regression analysis resulted in the following predictive formula:

(1) FVS=25.513.3∗log MAR+.

## 4. Discussion

Visual function is a complex construct, which integrates not only objective ophthalmic parameters but also functional aspects which are influenced by individuals’ motor skills, cognition, environment, and specific needs. Patients with similar impairments in visual acuity and visual fields may experience different levels of dysfunction and reduced quality of life. Several attempts have been made to develop tests evaluating patients’ functional vision. One such test included 17 timed instrumental activities of daily living tasks, which showed that lower visual acuity and smaller visual field were associated with longer completion times, even when adjusted for age [9]. Another is the “Visual Function Score,” a 51‐item test which incorporates elements of vision, ophthalmic findings, and ocular motility. This score was developed to quantify and monitor visual function in children, to aid in the rehabilitation of children with visual disorders [10]. These tests are complicated to administer and perform, require a considerable amount of time and skilled observers, and are also likely to be affected by patients’ motor and cognitive skills. Therefore, they have not been widely adapted clinically, and visual function is commonly evaluated by questionnaires, such as the VFI‐14 and VFQ‐25, which extrapolate an objective measure from patients’ subjective and variable self‐reporting [2, 4–6]. One purely objective score evaluating visual function was suggested by Bingöl‐Kızıltunç et al. based on response to threat, light, object, presence of fixation, duration of fixation, following light and objects in horizontal, vertical, oblique, and circular gazes, and optokinetic nystagmus [11]. However, this score was designed for children with visual disorders, as well as those with cerebral disorders, and is not applicable on a wide scale.

In the absence of an accepted method to evaluate visual function, this measure is currently estimated primarily from patients’ visual acuity, as well as their visual fields [2]. It does not consider specific individuals’ needs and abilities, which may differ due to age, cognition, and occupation, and does not determine their degree of dysfunction and quality of life. Therefore, there is an obvious need for a simple and pragmatic test to evaluate visual function, especially in modern society, in which different levels of disability are formally recognized by governments, insurances, and other agencies and are used to determine patients’ rights and benefits.

The 10 test items included in the present study simulate daily activities of modern life which require vision to complete, and the ability and time to complete them reflect functional vision. Overall, the test was simple to administer and perform, and its score also reflected participants’ motor and cognitive skills. As illustrated in Figure 1, participants with similar visual acuity had variable FVS scores, and participants with differing visual acuities in the different groups could achieve similar FVS scores. This indicates that an individual’s score reflects his or her level of visual function and considers their actual abilities—not merely their visual acuity.

The study results showed differences in the overall success scores between groups. Although even participants in Group 4 could complete most tasks, they took significantly longer to do so. Mean times to complete each task were significantly different between groups, and these differences highlight the differences in visual function. We note that not all participants had 20/20 vision in both eyes, making their mean time results a more realistic measure of the overall patient population. Although a FVS score of under 10 is possible (if a specific individual is faster than the mean), it is likely to assume that FVS scores between 10 and 15 represent full normal visual function. Higher FVS scores correspond to increasing levels of visual functional impairment.

FVS scores were strongly associated with visual acuity in the participants’ better eyes, and linear regression analysis resulted in the following predictive formula: FVS = 25.5 × logMAR + 13.3. However, it is clear that FVS is not only dependent on visual acuity, as scores can overlap between participants in different groups. Figure 2 illustrates the distribution of individual results around this line. This supports the concept that the FVS is a more robust assessment of visual function, and the study results show that the FVS test can be used to determine patients’ level of vision‐related disability.

We acknowledge that the test we designed for this study has not been previously validated. However, the concept of using timed functional tasks to assess functionality has been used previously to evaluate visual function [9, 10]. These tests were also used to evaluate the influence of vision aids in patients with low vision [12], as well as the effect of gene therapy in patients with RPE65 mutation–associated, inherited retinal dystrophy [13, 14]. Our results indicate that such a test can be applied to assess patients’ visual function and used to monitor progression or to determine rights and benefits. The FVS can easily be modified to include other visual function tests, adapted to different populations or settings, and validated with a larger cohort. We note that the FVS does not consider specific pathologies that cause visual impairment, as it was designed to assess visual function based on visual acuity regardless of its cause. We also acknowledge that patient motivation may influence the results of this test and that performance may be intentionally lower or slower if patients are motivated to gain formal recognition or monetary benefits due to their visual handicap. However, we believe a skilled and experienced tester may be able to identify these cases. Additionally, we note that although the sample size was not large, it was sufficient to achieve statistically significant results. We also note that participants in Group 1 were younger than those in Groups 2–4 as a limitation, as they could have motor and cognitive skills that decreased the time it took to complete tasks compared to the older participants, regardless of visual acuity. However, we used their mean time as the reference point to score the FVS, assuming that this level of function represents full normal function. Another limitation was that visual field information was not included in the study. Nevertheless, the results for each task and the FVS scores are a measure of their functionality, accounting for visual acuity and visual fields, as well as additional aspects, such as motor and cognitive skills.

In conclusion, this study demonstrated the feasibility and applicability of a relatively simple test to assess visual function, which correlates with patients’ visual potential and provides a more comprehensive evaluation of their visual function. We believe it may be a useful tool for clinicians and for patients, especially those with low vision or undergoing rehabilitation. Additionally, it may be useful for official agencies, which can improve the identification of patients with lower rates of functionality and independence, grade their level of vision‐related function loss, and allocate funds to aid those who need it most.

## Funding

No funding was received for this work.

## Disclosure

The protocol for this study was approved by the Institutional Review Board of Meir Medical Center in adherence to the Declaration of Helsinki, and all participants gave informed consent.

## Conflicts of Interest

The authors declare no conflicts of interest.

## Supporting Information

Additional supporting information can be found online in the Supporting Information section.

## Supporting information


**Supporting Information 1** Video 1: A patient from group 1 completing a task in which she was asked to identify a specific medication among 4 similarly shaped packages, in 5 seconds.

**Supporting Information 2** Video 2: A patient from group 4 completing the same task, in 27 seconds.

## Data Availability

Data are available upon request from the corresponding author.
